# Retinal Vasculitis in a Patient With Mixed Connective Tissue Disease Following COVID-19 Infection: Correlation or Coincidence?

**DOI:** 10.7759/cureus.26365

**Published:** 2022-06-27

**Authors:** Salil Mehta, Niharika Gill

**Affiliations:** 1 Ophthalmology, Lilavati Hospital, Mumbai, IND; 2 Rheumatology, Lilavati Hospital, Mumbai, IND

**Keywords:** systemic inflammatory and autoimmune disease, fundoscopy, covid-19, retinal vasculitis, mixed connective tissue disease

## Abstract

We report the systemic and ophthalmic findings in a female patient with mixed connective tissue disease (MCTD) who subsequently developed retinal vasculitis following coronavirus disease 2019 (COVID-19) reinfection. The patient was a known case of MCTD maintained in remission on immunosuppressive treatment. She subsequently developed retinal vasculitis with areas of capillary non-perfusion in the right eye. This was a finding not seen previously. She was started on an enhanced immunosuppressive regimen along with scatter laser photocoagulation. COVID-19 has been reported to lead to the development of autoimmune disease, both de novo as well as the worsening of pre-existing disease. The onset of retinal vasculitis may potentially be due to a post-COVID-19 exacerbation of her pre-existing MCTD. Physicians should be aware of this possibility and screen for the same.

## Introduction

Retinal vasculitis is a rare finding in patients with mixed connective tissue disease (MCTD). We report the systemic and ocular findings in a female patient on maintenance immunosuppression who developed retinal vasculitis following coronavirus disease 2019 (COVID-19) reinfection.

## Case presentation

A 56-year-old female, fully vaccinated against COVID-19 infection, was referred for a routine ophthalmic evaluation from the rheumatology clinic.

She was a known case of hypothyroidism on oral thyroxine replacement therapy (100 mcg daily). MCTD/overlap syndrome had been diagnosed four years earlier (February 2018). At that time, she was found to have an elevated serum creatinine (1.37 mg/dl; normal range: 0.6-1.2 mg/dl) during a routine checkup and was subsequently investigated. A 99M-Technicium renal scan revealed bilateral renal dysfunction as suggested by the reduced glomerular filtration rate (GFR) values (left kidney: 50.34 ml/min; right kidney: 12.04 ml/min) with a relative function of 80.69% on the left side and 19.31% on the right side. Immunological tests revealed a positive antinuclear antibody [ANA; by immunofluorescence (IFA)] test result with a speckled pattern (estimated titer: 1:320; moderately positive). Tests for anti-neutrophil cytoplasmic antibody (ANCA), both p-ANCA and c-ANCA, were negative. Further immunological testing returned negative results for anti-double strand DNA (ds-DNA), SSA-RO IgG, SSB-La IgG, Sm antibody IgG, Jo-1 antibody IgG, centromere antibody IgG, scl-70 IgG. Significant findings included positive test results for RNP-Sm antibody IgG (21.24 RU/ml; negative <20 RU/ml) and U1-snRNP 68 KDa IgG (5.4 RU/ml; negative <5; equivocal 5-10; positive >10 RU/ml).

At that time, a routine ophthalmic evaluation consisting of visual acuity, the slit-lamp exam for the anterior segment, an undilated fundus exam, and an assessment for a dry eye state were normal. She was started on an immunosuppressive regimen consisting of oral hydroxychloroquine (HCQS) 300 mg once daily and oral azathioprine 50 mg once daily. She subsequently went into remission without developing any further symptoms.

**Figure 1:**
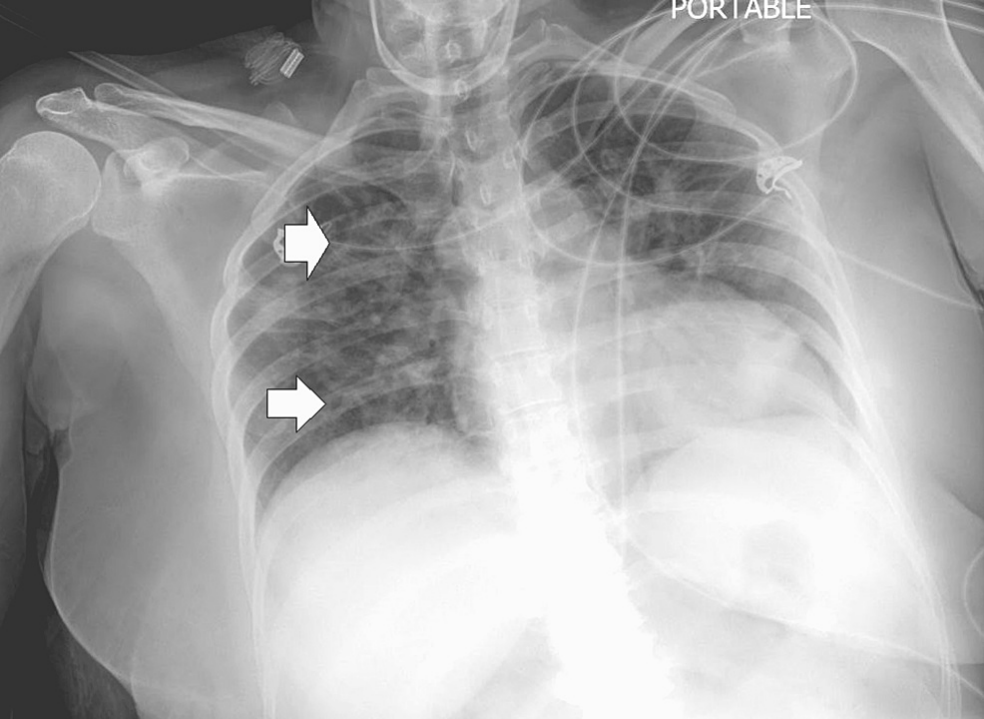
Chest X-ray showing multiple shadows across the upper, mid, and lower zones bilaterally. The arrows point to the lung field shadows on the right side

In August 2020, she developed fever, cough, and dyspnea, and was subsequently investigated and diagnosed with COVID-19 infection based on a positive reverse-transcriptase polymerase chain reaction (RT-PCR) test. A chest X-ray revealed multiple shadows in the upper, mid, and lower zones bilaterally (Figure 1), and these findings were evaluated by a CT scan that revealed multiple areas of ground-glass haziness bilaterally with a severity score of 5/25.

She was admitted and treated with a standard regimen consisting of intravenous remdesivir 100 mg once daily for six days, intravenous methylprednisolone 40 mg twice a day, broad-spectrum antibiotics, oral ivermectin 12 mg for three days, and subcutaneous Clexane (60 units twice a day). She was advised to continue the oral azathioprine and HCQS during the admission.

In November 2021, she underwent a detailed ophthalmic evaluation (anterior and posterior segment) and all findings were within normal limits. In January 2022, she had reinfection with COVID-19. She was managed with a single bolus of monoclonal antibodies (intravenous casirivimab 600 mg and imdevimab 600 mg) with the continuation of her maintenance immunosuppression.

**Figure 2:**
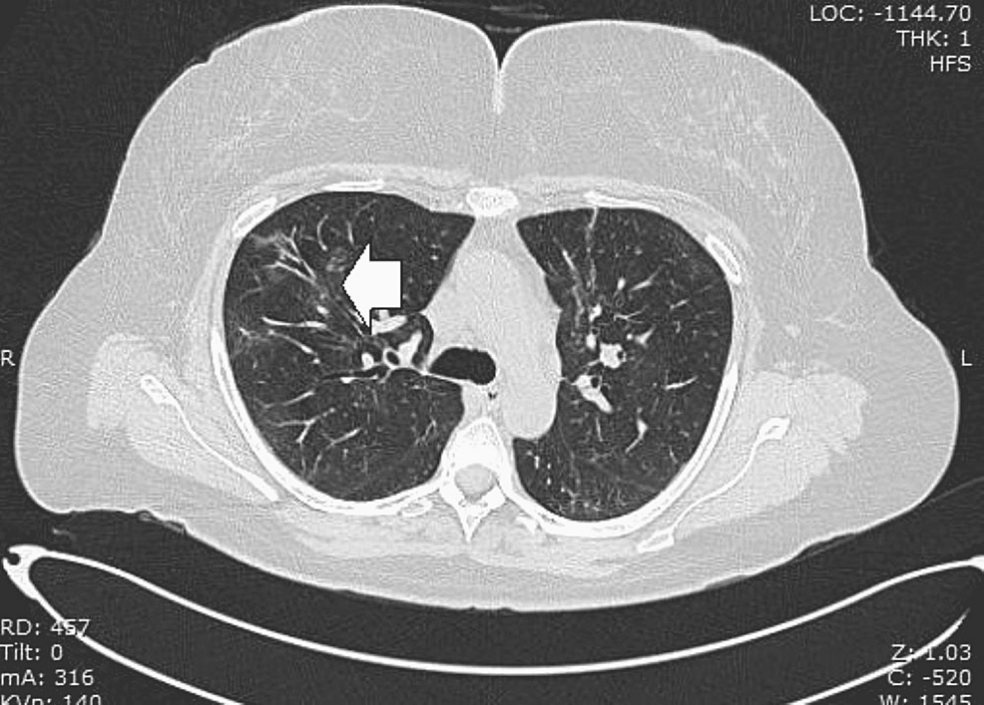
CT scan of the chest showing multiple ground-glass opacities scattered throughout the lung fields consistent with resolving COVID-19 pneumonia. The arrow points to an area of ground-glass opacity CT: computed tomography; COVID-19: coronavirus disease 2019

Two months later (March 2022), she was reviewed by the rheumatologist who ordered baseline blood work and a follow-up chest CT scan, which suggested resolving COVID-19 pneumonia (Figure 2). The rheumatologist requested an ophthalmic evaluation.

**Figure 3:**
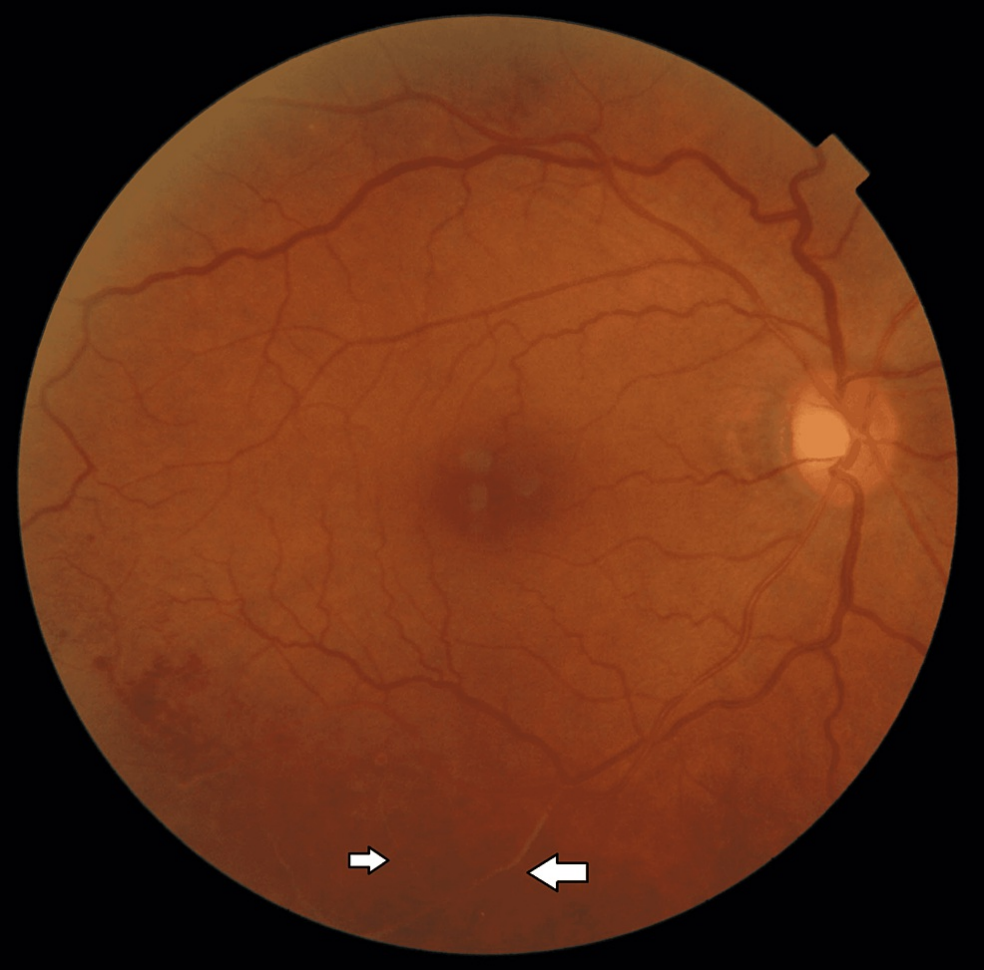
Fundus photograph of the right eye showing areas of narrowing and vasculitis (white arrows)

**Figure 4:**
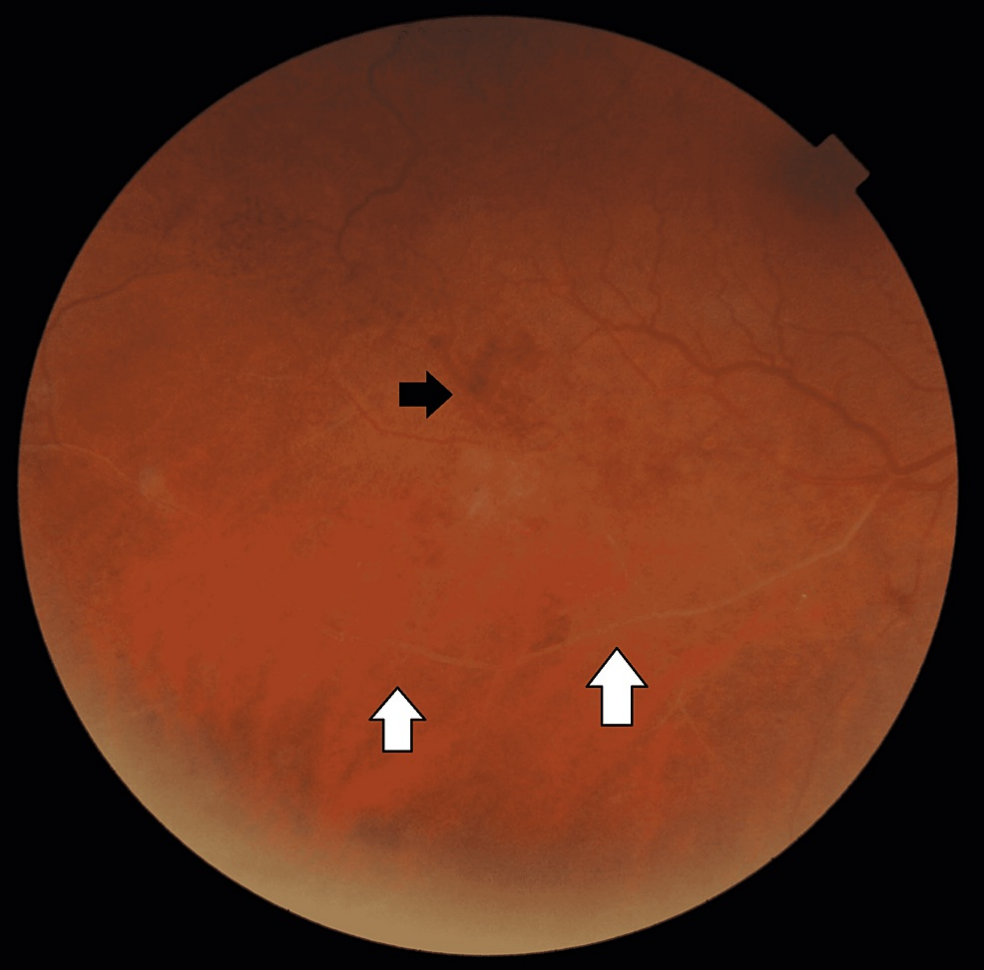
Fundus photograph of the inferotemporal quadrant of the right eye showing vasculitis (white arrows) and intraretinal hemorrhages (black arrow)

She was evaluated again in April 2022. On examination, she was asymptomatic, with her visual acuity being 6/6 and N6 in either eye. Extraocular movements, slit-lamp examinations of the anterior segment, and intraocular pressures were normal in both eyes. Dilated fundus examination revealed extensive vaso-occlusive disease, primarily venular, in the inferotemporal quadrant consistent with retinal vasculitis, intraretinal hemorrhages, and collateral formation in the right eye (Figures 3, 4). The left eye was normal.

**Figure 5:**
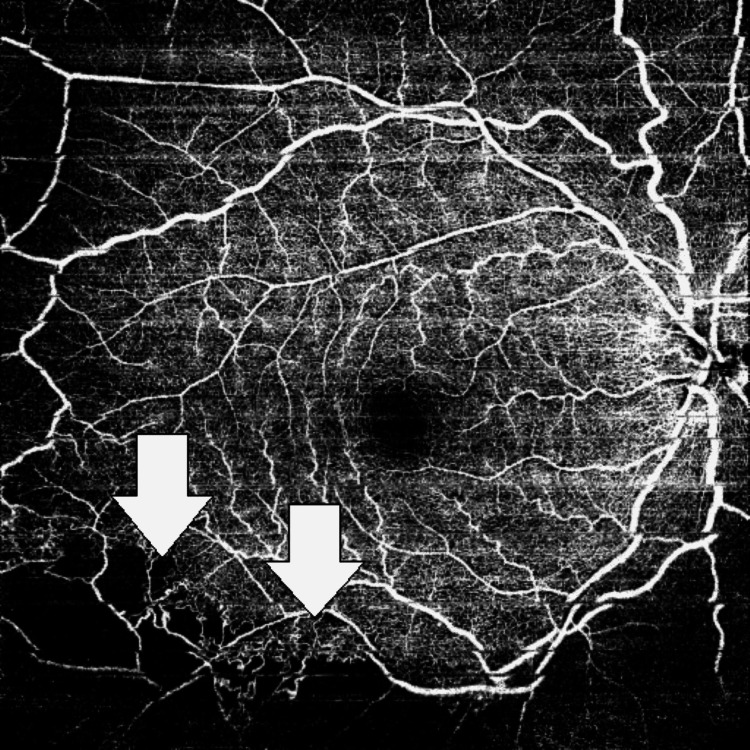
OCTA image showing areas of capillary dropout (ischemia) within the superficial capillary network (white arrows mark the boundaries of the ischemic areas) OCTA: optical coherence tomography angiography

The patient underwent an optical coherence tomography angiography (OCT/OCTA), which revealed significant areas of capillary dropout in the inferotemporal quadrant associated with the areas of vascular occlusion (Figure 5). The use of OCTA scan precludes the assessment of vessel wall inflammation, which is best appreciated in a fluorescein angiographic study.

She was initiated on an enhanced immunosuppressive regimen consisting of intravenous soluble methylprednisolone (500 mg daily for three days) followed by oral prednisolone (1 mg/kg/body weight) and an increase in the dosage of azathioprine (50 mg) from once to twice daily. A week later, she underwent scatter laser photocoagulation to the areas of capillary non-perfusion.

## Discussion

We discuss the systemic and ophthalmic findings in a patient with MCTD who was reinfected with COVID-19. Her previous ophthalmic examinations, the first one four years and the second five months prior to the presentation, were normal, possibly due to her use of immunosuppressives. On her last examination, there was evidence of retinal vasculitis and associated areas of capillary non-perfusion without any additional systemic findings following COVID-19 infection. This suggested other organ involvement due to disease exacerbation.

MCTD is a syndromic entity with mixed features of various autoimmune diseases including systemic lupus erythematosus (SLE), polymyositis/dermatomyositis (PM/DM), and systemic sclerosis (SSc). The diagnosis is usually based on the detection of high titers of anti-U1small nuclear (sn) anti-ribonucleoprotein (anti-RNP) antibodies [1].

Systemic vasculitis has infrequently been reported in patients with MCTD, with current estimates suggesting an incidence of 10-30%. In one large study of 112 MCTD patients, 11 patients were detected with vasculitis, largely cutaneous [2]. Retinal vasculitis has only rarely been reported and the underlying causative mechanisms remain undefined. We performed a search across the PubMed database using various combinations of the keywords “mixed connective tissue disease AND retina/retinal vasculitis” and were able to find only a limited number of case reports that described retinal vascular involvement.

Margolis et al. have described the findings in and management of a 46-year-old lady who was under treatment for MCTD and who presented with bilateral painless loss of vision. Fundus findings included optic disc edema, venous dilatation and tortuosity, and intraretinal hemorrhages bilaterally, all suggestive of occlusive retinal vasculitis. These findings were confirmed angiographically in the form of optic disc leakage, venous vessel wall staining, and areas of capillary non-perfusion. The patient was subsequently started on oral cyclophosphamide treatment and responded well. She subsequently developed macular edema in the left eye with a visual reduction to 20/400. It spontaneously improved to 20/80 in that eye and, due to the presence of persistent macular edema, she had an intravitreal injection of bevacizumab, which led to the improvement of the vision to 20/60 [3].

Kim et al. have reported the fundus and systemic findings in a 35-year-old woman who presented with acute visual loss in the right eye (20/100). Fundus lesions included retinal hemorrhages, venous tortuosity, cotton-wool spots, and macular edema consistent with a clinical diagnosis of central retinal vein occlusion (CRVO). Angiography studies detected areas of capillary nonperfusion and venous leakage during the later phases. She underwent a comprehensive therapeutic regimen consisting of intravitreal triamcinolone acetonide and oral immunosuppressants (prednisolone, cyclophosphamide, and methotrexate). Despite these measures, she continued to deteriorate and subsequently developed foveal capillary non-perfusion with a reduction of her vision to counting fingers [4].

Mimura et al. have described the findings in a 53-year-old Japanese woman with previously diagnosed MCTD, with recurrent retinal vasculitis and vitreous hemorrhages unilaterally. Three years later, she presented with reduced vision in the same eye (counting fingers) and was confirmed to have retinal and vitreous hemorrhages and retinal infarction. She was started on oral prednisolone (20 mg) with subsequent improvement of vision to 20/20 [5].

Dodds et al. have documented the clinical findings in a 16-year-old female patient with a systemic diagnosis of MCTD, who presented with central retinal vein occlusion. She was subsequently treated with plasmapheresis with the resolution of the disease [6].

COVID-19 is a widespread infection of recent origin that is caused by the severe acute respiratory syndrome coronavirus 2 (SARS-CoV-2). Patients commonly present with pneumonia, acute respiratory distress syndrome, dysrhythmias, myocarditis, or thromboembolic events. Immune dysregulation leading to a hyperinflammatory response is common in severely ill patients and manifestations include cytokine storm and macrophage activation syndrome. Potential mechanisms include alterations in the cytokine milieu, impaired regulatory responses that govern normal macrophages/monocyte activation, impaired natural killer cell function, reduced numbers of CD4+ and CD8+ T cells, and neutrophilia [7].

Viral infections are known to play a significant role in the development of autoimmune phenomena in patients with impaired immune regulation. Several autoimmune diseases have been reported in survivors of influenza, Ebola, and chikungunya in subsequent months. Similarly, there has been significant interest in the development of autoimmune phenomena following COVID-19 infection. This includes both the development of new autoimmune diseases as well as the exacerbation of previously diagnosed states. Numerous reports have described the onset of systemic sclerosis [8], lupus nephritis [9] as well as the exacerbation of multiple sclerosis [10], psoriasis [11] as well as autoimmune thyroiditis [12].

Numerous theories have been postulated to explain this phenomenon. These include (a) molecular mimicry: the development of humoral and cellular autoimmunity may occur as a result of a cross-reacting epitope between SARS-CoV-2 and the host and the potential loss of self-tolerance; (B) activated lymphocytes and macrophages may accumulate at tissues and induce bystander killing of adjacent healthy cells due to pro-inflammatory cytokines; and (C) inadequate or ineffective viral clearance may lead to the persistence of viral antigens and consequent polyclonal activation.

Another possibility is the development of neutrophil extracellular traps (NETs). These consist of an extracellular meshwork of nuclear chromatin fragments along with antibacterial proteins and enzymes. NETs are now thought to be responsible for the development of vessel wall inflammation and occlusive vasculitis in a variety of conditions [13].

Our patient had exacerbation of MCTD following COVID-19 infection. Retinal vasculitis is a reported albeit rare finding in MCTD and this development could well have been part of a natural worsening of her disease over the last few months. While her blood work showed no significant abnormalities, she was not assessed for hyperinflammatory markers (serum D-dimer, serum IL-6). Nevertheless, the temporal association between COVID-19 infection in January 2022, her previously normal ophthalmic evaluation (November 2021), and the subsequent development of retinal vasculitis (April 2022) suggests the possibility that COVID-19 infection induced an exacerbation of the MCTD that led to new-onset retinal vasculitis.

## Conclusions

Retinal vasculitis is an uncommon finding in the systemic vasculitis spectrum and has only rarely been reported in MCTD. The findings we describe may represent a natural progression of the disease or may have been influenced by COVID-19 infection. COVID-19 infection has been associated with the development of new autoimmune diseases as well as the exacerbation of pre-existing diseases. The timeline suggests a potential role of COVID-19 infection in the new-onset retinal vasculitis in this case. Physicians should be aware of post-infectious worsening of pre-existing autoimmune disease with the potential for ocular involvement and should screen for the same.
